# Study protocol for EthnoPRISE: an ethnographic study of peer sexual harassment in school

**DOI:** 10.1186/s40359-026-04361-4

**Published:** 2026-03-28

**Authors:** Kristina Holmqvist Gattario, Hannah Hallén, Therése Skoog, Robert Thornberg, Carolina Lunde

**Affiliations:** 1 Department of Psychology, University of Gothenburg, Box 500, Gothenburg, 40530 Sweden; 2 Department of Behavioural Sciences and Learning, Linköping University, Linköping, 58183 Sweden

**Keywords:** Peer sexual harassment, Ethnographic design, Adolescence, School, Peer relations

## Abstract

**Background:**

Despite children and adolescents having the right to an education free from sexual harassment, peer sexual harassment remains a common part of everyday school life. While peer sexual harassment is known to have many adverse consequences for students, there is a lack of research investigating students’ own perspectives on the matter. Additionally, there is little exploration of the sequence of events leading up to incidents of such harassment, and of the reactions that follow by those involved. Therefore, the primary objective of the EthnoPRISE project is to understand peer sexual harassment based on how it is expressed in students’ everyday school lives across adolescence, and how it is perceived, interpreted, and experienced by the students themselves.

**Methods:**

The EthnoPRISE project has a short-term ethnographic design and will include field observations, informal conversations, and focus group interviews. Three separate sub-studies will be conducted, each focusing on a different age group. Drawing from a developmental contextual perspective, data will be collected in Grade 5 (11–12 years), Grade 8 (14–15 years), and the second year of high school (17–18 years). One or two classes will be observed per age group, adding up to approximately 25 weeks of total observations across grades. In addition, at least 10 focus group interviews will be conducted per grade (totaling approximately 120 participants). We will use qualitative analyses, including reflexive thematic analysis and constructivist grounded theory, to analyze the data.

**Discussion:**

The EthnoPRISE project has the potential to provide novel insights into students’ experiences of peer sexual harassment in school. The findings from the current project may contribute to a deeper understanding of the complexity surrounding peer sexual harassment in schools and to inform more effective and developmentally appropriate measures to prevent it. Further, the project will give students the opportunity to express their perspectives on peer sexual harassment including their opinions on what should be done to reduce the problem.

**Supplementary Information:**

The online version contains supplementary material available at 10.1186/s40359-026-04361-4.

According to the Swedish Education Act [50], children and adolescents have the right to an education free from sexual harassment (SH). Despite this, peer sexual harassment (PSH) – unwanted sexual attention from peers – remains a common part of everyday school life [27, 64]. Students’ involvement in PSH is related to a range of adversities, including emotional problems, substance use, lower school belonging, and poorer academic engagement [26, 36, 45, 64], calling for early intervention. While the literature on PSH is growing, the existing research is mainly based on quantitative approaches that overlook how students themselves perceive, interpret, and experience what research and policy documents define as SH.

Additionally, there is a lack of knowledge about the expression and course of events of PSH in the complex reality where it occurs (most often at school; [17]). To understand what underlies the reportedly high prevalences of PSH in school and to prevent the problem, more knowledge is needed about these situations, both based on young people's voices and by observing students' everyday lives in school. The current project, EthnoPRISE (an Ethnographic Study of Peer Relations in School from an Ecological Perspective), therefore aims to understand PSH from students’ own perspectives, including how they perceive, interpret, and experience it, as well as how it is expressed in their everyday life at school.

In the Swedish Discrimination Act [49], SH is defined as a “behavior of a sexual nature that violates someone’s dignity. In addition to comments and words, it can also involve, for example, groping or intrusive staring.” In the research literature, SH is often defined as “unwanted sexual attention” [39], involving for example being exposed to uninvited sexual comments, homophobic and misogynistic name-calling, unwanted grabbing and touching of private body parts, and being sent unwelcome sexual pictures through digital means [3, 27]. These definitions emphasize the victim's perspective (e.g., the victim decides whether the attention is unwanted) when assessing whether an act qualifies as SH [41, 52]. In line with this, the current project focuses on how students themselves perceive, interpret, and differentiate behaviors as either SH or not. By delving into students’ perspectives on such incidents, the current project aims to broaden our comprehension of PSH, extending beyond the academic and legal definitions.

In our ongoing research project PRISE (Peer Relations in School from an Ecological Perspective; [52]), based on annual questionnaires to a large sample of students (*N* = 997), we have found that already at the age of 10, as many as half of the students had been called “slut” or “gay” or had been groped by a peer [64]. As students get older, these experiences are even more frequently reported [17, 46]. In the light of academic and legal definitions, situations of homophobic and misogynistic name-calling or groping may be considered typical examples of PSH. However, the limited research examining PSH from students’ perspectives, indicates that they do not necessarily think of such situations as PSH and that they are often uncertain of what behaviors constitute PSH [23, 35, 56, 59]. A mixed-methods study involving 638 students aged 13–17 used a survey of PSH alongside 18 focus-groups with 119 students exploring their perceptions of these behaviors [56]. Findings showed that two-thirds of survey respondents reported experiencing verbal/visual or physical PSH within the past three months. However, focus group findings indicated that several students were unsure whether certain behaviors (e.g., being touched, grabbed, or pinched) qualified as harassment. Further, when asked to classify behaviors (e.g., being made to kiss someone or receiving sexual comments about one's appearance/body) as “OK”, “Not sure”, and “not OK”, participants expressed uncertainty about where the line falls between the acceptable and unacceptable behaviors. Participants' uncertainty about what constituted PSH largely depended on contextual factors, including the relationships between those involved, the level of pressure exerted by the instigator, and the perceived intent of the instigator(-s). Other research similarly highlights young people’s uncertainty in defining and evaluating PSH. An interview study with 7- to 12-year-old girls found that participants did not view all instances of PSH as equally problematic, rather their judgments depended heavily on contextual factors, including the relationship between those involved and the instigators' perceived intents [23]. Further, a qualitative exploratory study using group discussions with seventh graders (aged 13) reported that the concept of SH was difficult for participants to grasp and distinguish from other behaviors, such as being touched by a nurse during a medical examination [35]. Arguably, these findings highlight the complexity of PSH and point to the need for young people’s perspectives in research that concerns them (see e.g., [44]). The importance of contextual factors in young people’s perspectives also underscores the need for a deeper understanding of how situations of PSH unfold in students’ everyday lives—something that cannot be fully captured through self-reports or interviews alone.

## Ethnographic research with children and adolescents

Ethnography is a qualitative research method involving extended fieldwork in participants' natural environment, often combined with some form of interviews (e.g., informal in-the-moment interviews and formal interviews, in group or individually; [24, 29]). As such, it is especially suitable for studying students’ everyday lives and their perceptions of frequently occurring events. Further, ethnography is an approach that is participant-centered and emphasizes reflexivity, immersion, and context. Because children and adolescents have their own culture of communication, ethnography is particularly beneficial in capturing their experiences and culture as it allows for dialogue and reflexivity [14, 19]. Also, as PSH is reported to occur in all areas of schools (e.g., classrooms, hallways, school yards, cafeterias, and changing rooms [1, 2, 15], as well as in the presence of school personnel [25], ethnography is likely an effective approach of capturing how PSH is expressed in students’ everyday lives.

A few studies have used ethnographic methods to explore peer victimization and bullying in schools (e.g., [22, 40, 54, 61]), but there is a scarcity of ethnographic research investigating PSH specifically. To date, the study most similar to the current project is Renold’s [48] more than 20-year-old ethnography, which examined physical and verbal forms of heterosexual, homophobic, and heterosexist harassment among students in their final year of primary school (aged 10–11) in England. Based on one year of fieldwork at school, but mainly exploratory unstructured group interviews with the students, Renold documented a range of behaviors, including instances of verbal heterosexual harassment usually centered around the girls’ sexual status (e.g., boys calling girls “slag” or “bitch”). Physical forms of PSH (boys victimizing girls) were less common but were reported by some girls (e.g., being punched in the breast). Interestingly, while Renold first positions boys as the primary instigators of SH, she also describes “predatory girls” who “found their positions as ‘girlfriends’ and their new-found sexual knowledge as particularly powerful” (p. 423). These girls were described as sexually intimidating “subordinate boys” and/or were physically violent towards their boyfriends. Finally, Renold documented instances of homophobic name-calling, and bullying behaviors towards those who did not align with norms of masculinity and femininity. The absence of ethnographic studies specifically addressing PSH since Renold’s study underscores a clear gap in the existing literature. This gap emphasizes the importance of the current project as it aims to address a notable lack of student-centered research exploring PSH at school.

## Theoretical frameworks

The current project will explore PSH from a developmental-contextual model [33] and a gender perspective (e.g., [11]). While various models and perspectives aim to explain PSH, these two have been selected to guide the project’s overall direction. Since the ongoing analysis of field data may highlight other relevant theories and concepts, we remain open to incorporating additional theories as the project progresses.

### Developmental contextual model

The developmental contextual model [33] is a framework used in psychology to understand human development and how it is affected by influences such as interpersonal relationships, societal factors, and cultural norms. The model offers a useful framework for studying PSH among adolescents as it situates these behaviors within broader developmental processes (e.g., puberty and cognitive and emotional development) and the social contexts (e.g., the class, the school, social media, and society at large) in which adolescents interact (see e.g., [38]). According to this model, how adolescents perceive, interpret, and experience PSH may be influenced both by their current developmental stage and by their exposure to social and environmental factors, such as norms, power dynamics, societal structures, and cultural attitudes [7, 38].

Childhood and adolescence encompass several developmental stages and processes, with puberty arguably being the most crucial process to consider in the context of sex and sexuality [51]. The onset of puberty begins around ages 8–14, with girls, on average, entering puberty 1.5 years earlier than boys [31, 42]. During puberty, adolescents begin to engage in sexual behaviors and learn to express their sexual interests and desires in a socially acceptable way [4, 20]. Still, emotional and cognitive abilities, including self-regulation and the ability to take others’ perspectives, continue to develop during the following years [6]. Consequently, instances of PSH may, especially during puberty, emerge as misguided attempts to express sexual interest [15].

Further, and in line with the developmental contextual model, how adolescents perceive, interpret, and experience PSH needs to be understood in relation to their social context. What adolescents perceive as normal or associated with higher or lower status is directly related to the norms they are exposed to in their family, peer group, school, or society [11]. Norms related to gender, sex, and sexuality may be particularly influential when it comes to PSH (see also Gender perspective). From a cultural point of view, Sweden, the cultural context where the current project will be carried out, is often considered more liberal when it comes to attitudes toward sexuality or sex [34, 53]. Also, Swedish schools are obliged to integrate education concerning sexuality, puberty, consent, gender equality, and healthy relationships into the teaching of various school subjects (Swedish National Agency for Education, 2025). At the same time, research shows that gender stereotypical norms related to sex and sexuality—such as girls being more shamed when expressing and exploring their sexuality—were present in a sample of Swedish adolescents too [12]. These, to some extent, contradictive cultural norms may influence how Swedish adolescents perceive and think about PSH.

Informed by the developmental contextual model, the current project will examine PSH in adolescents of different ages; at early-, mid-, and late adolescence. Accordingly, the participants will represent different developmental stages, which may shape both the expression of PSH and the ways in which it is perceived, interpreted, and experienced by the participants. Research shows that PSH tends to escalate as adolescents grow older [17, 46], and older adolescents seem to be more accepting toward PSH [28]. Further, there may be developmental differences in the types of PSH that are most frequent. For example, in a sample of 10 to 15-year-olds, Espelage et al. [17] found that younger students were less likely to report both verbal and physical forms of PSH but reported a higher rate of being “spied on” by their peers in changing rooms. This variation in experiences and attitudes may be attributed to differences in developmental stages, particularly in relation to pubertal development, peer norms, and cognitive and emotional understanding of occurrences of PSH. Regardless, the variation underscores the need to examine how PSH evolves and takes shape throughout different developmental stages within the school context.

### Gender perspective

Closely aligned with the developmental contextual model, we will use a gender perspective (e.g., [11]) to understand potential gender-based differences and similarities in power and social status related to PSH. Brown et al. [11] proposed that children’s social contexts (parents, school, peers, and the media) contribute in various ways to the endorsement of stereotypical gender norms (girls as passive and pretty and boys as aggressive and dominant). As children develop into adolescents, they suggest that these norms evolve into sexualized gender stereotypes (i.e., girls as sexualized objects for boys, boys as sex-focused and assertive) potentially reinforcing and normalizing PSH behaviors [11]. Further, PSH can operate as a form of gender policing, for example, by using derogatory words such as “slut” or “whore” to police girls’ sexual behaviour [15], or, among boys, through homophobic name-calling [18, 21]. Research has shown that gender-nonconforming boys are especially targeted for homophobic name-calling [65], and that other gender-nonconforming boys may use homophobic name-calling to try to avoid being targeted themselves [57]. It is important to highlight that several findings (e.g., [39, 48, 64]) refute the classical notion that PSH is one-directional, that is, that boys perpetrate, and girls are the victims of PSH. Thus, we will not define PSH as boys’ aggression towards girls [52].

It is arguably essential to differentiate between same-gender and cross-gender PSH when discussing and researching the phenomenon. Cross-gender PSH, which is believed to be related to pubertal onset and sexual maturation, increases throughout adolescence, potentially as a result of misguided attempts to show sexual interest [15, 39]. In contrast, same-gender PSH has been suggested to be motivated by attempts to assert dominance and gain social power [39, 64]. This form of harassment often manifests through behaviors such as homophobic name-calling, hazing rituals, sexual rumor spreading, and gender-related insults. In this project, both same-gender and cross-gender PSH will be considered.

## Aims and objectives

Based on the identified gaps of knowledge in the field, the primary objective of the EthnoPRISE project is to understand PSH based on how it occurs in students' everyday lives at school and how students themselves perceive, interpret, and experience it. The project allows for a deeper understanding of students’ perspectives on PSH, and offers insights that cannot be captured by the commonly used structured self-report measures, which are typically designed based on adults’ perceptions of PSH. Knowledge about how PSH occurs in students’ everyday life, and how students themselves experience and describe it is important as it can inform actions against PSH in schools. For example, knowledge about whether students describe PSH in ways that are distinct from adults’ definitions, or if students of different ages describe PSH in different ways, should be considered when developing interventions. Further, expanding the understanding of the sequence of events of PSH can allow for the development of more accurate measures that could be used preventatively.

The project aims to answer the following research questions:

1. How is PSH expressed among students in early-, mid-, and late adolescence?
2. How do students in early-, mid-, and late adolescence perceive, interpret, and experience PSH at school?
3. Which processes/courses of events lead to or counteract situations of PSH at school among students in early-, mid-, and late adolescence?
4. Which measures against PSH should be prioritized according to students in early-, mid-, and late adolescence?
5. What possible differences and similarities are there regarding the above questions depending on students' different ages?
6. How does students' way of understanding PSH relate to how it is defined in the scientific literature and school guidelines?

## Methods

### Design

To answer the research questions, we will use a short-term ethnographic design, including observations, informal conversations, and focus group interviews with students in early-, mid-, and late adolescence. Short-term ethnography involves researchers spending a concentrated, intensive period in the field, in contrast to classic ethnography, where fieldwork may span several months or even years [10, 47]. We will adopt this approach to allow for observations across multiple grades and schools, something that would not have been feasible within the project's budget and time constraints otherwise. The project will run over four years, with data collection conducted during the first three years. We will conduct three separate sub-studies, each one focusing on a distinct age group. Observations and interviews will be conducted in Grade 5 (project year 1), Grade 8 (project year 2), and the second year of high school (project year 3). The Swedish school system comprises three years of middle elementary school (Grades 4–6, ages 10–12), three years of junior high school (Grades 7–9, ages 13–15), and three years of high school (ages 16–19), hence the targeted grades represent the grade in the middle of these periods.

### Participants and procedure

We will use SALSA, an analytical tool provided by the Swedish National Agency for Education, to select a sample of schools in southern Sweden whose student populations most closely reflect the demographic profile of the average Swedish school. We will contact principals of the selected schools and ask whether the school is interested in participating in the project. From these, one to two classes per grade will be selected per sub-study. For a class to be included in the study, a clear majority of the students must consent to participation (at least 60%). With about 20 students per class, we expect the total number of students across the three sub-studies to amount to approximately 120.

Detailed information letters and consent forms will be sent to students, teachers, and legal guardians of those students who are under the age of 15 (in accordance with the Swedish Ethical Review Authority's policy; [55]). Parental consent will be required and obtained for all participants under the age of 15 prior to asking the students to consent. The information for students will be adapted linguistically to suit each age group. The information letters will contain a general outline of the study’s purpose, information about potential risks and benefits of partaking in the study, as well as contact details for the researchers. Additionally, all participants will be informed of their right to withdraw from partaking in the study at any point in time, without providing any reason or explanation. Participants may also consent to participate in certain parts of the study (e.g., fieldwork) while not consenting to participate in other parts (e.g., the interviews). While teachers are not the focus of the current study, they will also be asked to consent to participation as they will inevitably play a part in situations that will be observed and recorded.

All participating classes will be thanked for their participation with a monetary reward equivalent to 170 euros. However, this reward will not be disclosed until consent has been obtained, and the data collection is completed to ensure that participation is voluntary and uninfluenced by the incentive. We expect participant recruitment to be completed by June 2027.

### Data collection

#### Fieldwork

Observations will be conducted during regular school hours with the aim of obtaining as unfiltered a view as possible of students' day-to-day encounters with PSH in school. We will observe each grade for at least 3 days per week for at least 6 weeks, and one to two classes per grade will be observed during this period (i.e., 5–7 weeks per class). We expect the total time for observation in all classes to amount to approximately 25 weeks (at least 75 school days). We will observe students throughout the entire school day, and observations will be recorded through field notes, which will be made into a clean copy and transcribed at the end of each observation day. Informal, context-driven interviews with students and teachers will be conducted throughout the observational period to clarify and deepen our understanding of events as they unfold. One to two researchers will be conducting the fieldwork.

The researchers completing the fieldwork will attempt to adopt a “least-adult role” [37], meaning that they will aim to minimize their contact with other adults at the schools, avoid reprimanding students, and participate in students’ activities when possible. However, while aiming to stay true to their role as researchers, they will also be prepared to intervene should they encounter any situation where a student is at risk of physical or psychological harm.

#### Focus group interviews

In addition to the observations and informal interviews, we will conduct focus group interviews to get a deeper insight into how students perceive, interpret, and experience PSH. Participants in the observed classes consent verbally if they wish to participate in these follow-up interviews.

We have chosen focus groups over individual interviews to better capture the interactive nature of students’ conversations about PSH. The use of focus group interviews may also balance the power asymmetry between students and adults, so that the number of students are more frequent than the number of adult researchers, and students may also support each other during the interviews. However, students will also be given the opportunity to participate in individual interviews if they prefer. The interviews will last approximately one hour and will be conducted during regular school hours on the school premises. Each focus group interview will be conducted with 4–5 participants per group (recommended number: 4–8 people; [32]). We will form groups in collaboration with teachers and students and pay careful attention to creating groups of peers that are comfortable with each other. At least 10 focus group interviews will be conducted per grade, totaling 30 focus group interviews and a total of approximately *N* = 120 participants. The researcher/s conducting the fieldwork will be the same ones conducting the interviews.

A preliminary interview guide can be found under Supplementary material. The interview questions are informed by the project’s research questions and the items in the Peer Sexual Harassment – Child scale [63], which was designed for assessing PSH from age 10 and onwards. To stimulate discussion, we will use example scenarios of potential PSH for the participants to reflect upon. We will also ask participants, at least the younger ones, to reflect on PSH relative to bullying, a concept that may be more familiar to them. Notably, it is often difficult for researchers prior to data collection to predict how to formulate interview questions that would be relevant to the observations and comprehensible to participants, especially considering that adolescents often have different communication norms than the researchers themselves [16, 19]. Therefore, the interviews will be carried out as an extension of the observations and will partly be informed by situations that have been observed. Further, we will conduct pilot interviews with participants of each age group to ensure that the interview questions are phrased in an age-appropriate and eligible manner.

In addition to focus-group interviews with students, we will invite teachers to participate in individual interviews to provide contextual insights into classroom dynamics and students' interactions. The teacher interviews will be informed by observations and conversations with students, as well as by a pre-formulated interview guide.

#### Classification and transcription of data

We will adopt an iterative approach to data collection and analyzes, by conducting fieldwork and parts of the analyses simultaneously. Observational and interview data will be reviewed continuously within the research team, thereby allowing the data to guide our ongoing fieldwork and data collection. When the observations and informal interviews are carried out, notes will be taken simultaneously, made into a clean copy and transcribed after each collection day to avoid recall bias. The focus-group interviews will be audio-recorded and transcribed verbatim, including environmental details such as laughter, tone of voice, and disruptive noise. The names of both the schools and participants will be pseudonymized. The focus-group interview data will be transcribed using Whisper, an openAI speech to text system. The Whisper-generated transcripts will then be refined through manual transcription of each interview. All data will be stored and handled within a secure data environment in accordance with the European Union’s General Data Protection Regulation (GDPR).

### Analyses

We will analyze the data from observations and interviews using appropriate qualitative methods, such as reflexive thematic analysis [9] and constructivist grounded theory [13]. Reflexive thematic analysis is used for identifying patterns and themes in qualitative data, for example, related to how PSH is expressed among students in early-, mid-, and late adolescence (RQ1) and how the students perceive, interpret, and experience PSH at school (RQ2). The reflexive part of the thematic analysis emphasizes the importance of making the researcher’s assumptions and expectations visible in the analysis [9]. Incorporating reflexivity at each stage of the research process is particularly important in this study, as we are aiming to investigate PSH from a student perspective and recognize that adults and researchers may hold conceptions of PSH that differ from those of the students [30, 56].

Furthermore, constructivist grounded theory [13] is used to explore social processes and understand how participants perceive, interpret, and engage within these processes. This approach could, for example, be used to examine the processes/courses of events that lead to or counteract situations of PSH at school (RQ3), or to examine possible age differences in how students interpret PSH (RQ5). Given that PSH is fundamentally a social process, and that grounded theory has been effectively utilized in prior research examining bullying in schools as a social process in relation to group and societal norms [60], we consider grounded theory to be an appropriate methodological choice for investigating PSH in a school environment.

### Reflexivity

Reflexivity is a crucial aspect of ethnographic research [19], which we will consider and carry out throughout the entire duration of the project. The researchers carrying out the observations and interviews will keep reflexive diaries alongside their field notes. Additionally, during the observational period, the EthnoPRISE research group will meet weekly or biweekly to discuss the findings and reflect upon the next stages of the project. Further, we recognize that the researcher’s personal characteristics—such as gender, ethnicity, culture, and age—inevitably influence the fieldwork [19]. Considering the recognition that all co-authors’ academic habitus [8] shape the analysis, co-authors' demographics and professional background are presented below, and reflected upon in greater detail in each sub-study.

All members of the research team are white Swedes. The first author (K.H.G.) is a 44-year-old female Professor of Psychology. H.H. is 26-year-old female PhD Student in Psychology. R.T. is a 57-year-old male Professor of Education. T.S. is a 48-year-old female Vice Dean and Professor of Psychology, and a C.L. is a female 46-year-old Professor of Psychology and Director of Studies. Apart from H.H., all authors each have 20 + years of experience in conducting research on peer relationships, peer victimization, and/or PSH.

## Discussion

In a research field that has primarily relied on quantitative self-report measures reflecting adult and academic definitions of PSH (e.g., [5, 59, 66]), the EthnoPRISE project offers a unique opportunity to gain insight into how PSH is expressed in students’ everyday lives at school as well as how students themselves experience and describe PSH. The project aims to advance knowledge of PSH by providing a deeper understanding of how it is expressed among students of different ages, and how students of different ages perceive, interpret, and experience PSH. By integrating student-centered and developmental perspectives, this project offers a novel contribution to the field and may serve as a foundation for future theory development in PSH.

The current project can also help inform how to address and intervene against PSH in schools. Interventions that are based on adult or theoretical/non-empirical understanding of PSH may fail if they do not properly capture the way that PSH is expressed in students’ everyday lives or the way that students understand PSH. For example, interventions that involve teaching bystander peers to intervene when they witness situations of PSH will need to carefully address peers’ interpretations of situations as PSH as well as their ambiguities in these interpretations (“are they joking or not?”; [43]). Interventions may also have as their primary target to try to change students’ attitudes to PSH to reduce PSH [58]. Such interventions could benefit from more knowledge about students’ understanding of PSH to begin with. Not least, the current project can help inform PSH interventions as it will highlight the measures that students themselves (and teachers) believe to be most important in addressing this issue.

The ethnographic fieldwork, which is novel within the field of PSH, can make an important contribution to the literature by providing detailed knowledge about the sequence of events leading up to PSH incidents. This knowledge may be helpful in identifying especially risky situations at school, as well as important interactions in the peer group prior to and during PSH that promote or discourage PSH. Further, being researchers in the field allows us to consider contextual and environmental factors (e.g., group dynamics, how teachers/adults in the school speak about/respond to such events), which we would not be able to access if we were to use other methodologies. The fieldwork may also help in gaining participants' trust and building rapport, likely enriching the focus group interview data. Being present before, during, and after the interviews will enable a deeper understanding of what is being expressed during the interviews, while also providing opportunities for students to elaborate or follow up on issues that arise.

We believe that the students participating in this project will feel that they are contributing to something valuable and will appreciate being asked to share their opinions and views on the matter. Furthermore, the EthnoPRISE project aligns with the United Nations Convention on the Rights of the Child [62], which emphasizes that children should be encouraged to express their views on matters that affect their lives (Arts. 3, 12, & 13). Arguably, children and adolescents’ participation in research that concerns them can be seen as an extension of this principle [44]. Additionally, the contributions and perspectives of early-, mid-, and late adolescents are essential for understanding their experiences and daily lives, as adults’ perspectives often differ significantly from those of young people [30].

## Supplementary Information

Supplementary Material 1.

## Data Availability

The data generated and analyzed during the current study is not publicly available due to the sensitivity of the questions but is available from the corresponding author on reasonable request.

## Abbreviations

EthnoPRISE: An Ethnographic Study of Peer Relations in School from an Ecological Perspective
PRISE: Peer Relations in School from an Ecological Perspective
PSH: Peer sexual harassment
SH: Sexual harassment
