# Supplementary material for: Study protocol for EthnoPRISE: an ethnographic study of peer sexual harassment in school
Source: BMC Psychol. 2026 Mar 28;14:489. doi: 10.1186/s40359-026-04361-4 (PMC13063441; doi:10.1186/s40359-026-04361-4)
Supplement: Supplementary file 1 — Supplementary Material 1. [file 40359_2026_4361_MOESM1_ESM.docx]

**Preliminary Interview Guide for Focus Group Interviews with Students**

The interview guide is informed by the study's research questions. It will also be informed by the field work regarding, for example, the types of situations and vignettes discussed during the interviews.

**(Short Introductory Part)**

**Sexual Harassment in Relation to Bullying**

- What do you think bullying is? Can you give examples of situations that you think are bullying?
- What do you think sexual harassment is? Can you give examples of situations that you think are sexual harassment? (remind students that there are no right or wrong answers)
- What are the similarities and differences between bullying and sexual harassment, in your opinion?
- What are your experiences of sexual harassment? Have you seen/heard anyone victimizing someone or being subjected to what you would define as sexual harassment at school, online, or at after-school activities? Do you think it happens to students of your age? Why/why not?

**Expressions of Sexual Harassment and Students' Perceptions, Interpretations, and Responses**

- Have you heard of middle/junior high/high school students hugging or kissing another student even though they did not want to? Can you give examples of when this happens? Why do you think people kiss or hug someone who doesn't want to? How do you know if someone wants to kiss or hug you? How do you feel if someone hugs or kisses you when you don't want them to?
- Have you ever heard rumours about other students' sexuality or for example about someone making out with/kissing another student (examples adapted to the age of the students)? What do you think about that? Why do you think such rumours are made up and spread? How do you think the person the rumour is about feels?
- Have you heard of students in middle/junior high/high school making comments or jokes about private body parts or sex? Can you give examples of when this happens? Why do you think people make jokes about private parts or sex? How do you feel when others make jokes about private body parts or sex? What would you do if someone joked about private parts or sex?
- Have you heard of middle/junior high/high school students touching another student's private body parts (e.g. penis, bum, breasts) without their permission? Can you give examples of when this happens? Why do you think students touch another student's private parts when they don't want them to? What do you do if it happens to you?
- Have you heard of middle/junior high/high school students showing or sending other students’ pictures or messages related to nudity or sex? Can you give examples of when this happens? Why do you think such pictures are shown or sent? How do you think the person who sees/receives the pictures feels? What do you do if you see or receive images of nudity or sex that you don't want to see?
- Have you heard of middle/junior high/high school students calling each other gay, fag, lesbian, or similar words? Can you give examples of situations where this happens? Why do you think they say these words specifically? How does it feel to be called gay, fag, lesbian, or similar words?
- Have you heard of students in middle/junior high/high school calling each other a dick, cunt, slut, or similar words? Can you give examples of situations where this happens? Why do you think they say those specific words? How does it feel to be called a dick, cunt, slut, or similar?
- Have you heard of middle/junior high/high school students calling each other words like slut/gay/cunt or making jokes about sex/private body parts on social media? Do you think there is a difference between saying/doing such things on social media compared to in person?
- Is there a difference between a friend saying/doing something (e.g. calling you a slut/gay or slapping your bum) compared to someone you might not know very well?

**What Can be Considered Sexual Harassment According to Students**

This part uses different vignettes describing various types of PSH as defined by researchers. Students will be asked to discuss whether the situations described are sexual harassment. The vignettes will be adapted to the age of the students.

*In this part of the interview, I will describe different situations to you. Then I will ask you questions about what you think and feel about these situations.*

- *William mostly hangs out with the girls and is good at dancing and horse riding. Some people in his class sometimes call him a faggot or gay*. Do you think this is sexual harassment? Why/why not? Why do you think they call William gay? How do you think William feels when this happens? What do the other students do when William is called gay or fag? What do you think William should do?
- *Kalle sees Stina and Ahmed coming out of a group room at the same time. Stina and Ahmed had been told to sit in the group room by a teacher, but despite this, Kalle spreads a rumour to the other students in the class that Stina and Ahmed were kissing in there* (age appropriate). Do you think this is sexual harassment? Why/why not? Why do you think Kalle does what he does? How do you think Stina and Ahmed feel? What do you think Stina and Ahmed should do?
- *Vincent is interested in Bella in the same class as him. He doesn't know if Bella is interested in him. One night at a school disco/party, he tries to kiss her, but she recoils as if she doesn't want to be kissed*. Do you think this is sexual harassment?  Why/Why not? Why do you think Vincent does what he does? What should you do if you want to show that you are interested in someone? How do you think Bella feels when this happens? What do the other students at the disco/party do when this happens? What do you think Bella should do?
- *Mira sends a “joke” picture of two naked guys to her friend Nour. Nour finds the picture disgusting and wishes Mira hadn't sent it*. Do you think this is sexual harassment? Why/why not? Why do you think Mira sent the picture to Nour? How do you think Nour feels when she receives the picture? What do you think Nour should do?

**Actions Against Sexual Harassment**

- How does the school deal with sexual harassment? What do the teachers or other adults at the school do when such situations occur? How do you think the school should deal with this? What do you think adults at the school should do if they see or learn that someone is being victimized?
- Is there anything else you would like to mention that I/we have missed?

The interview guide for teachers is based on the above questions but is adapted to capture a teacher's perspective on how students perceive, interpret, and experience situations that can be considered sexual harassment, as well as how teachers and schools work with these issues.
